# The Traveling Mind: Moderators, Mediators and Pathophysiology of Migration Psychosis

**DOI:** 10.1192/j.eurpsy.2024.1274

**Published:** 2024-08-27

**Authors:** K. Kahil, L. Daou, A. Chabbouh, E. Ghossoub

**Affiliations:** Department of Psychiatry, American University of Beirut Medical Center, Beirut, Lebanon

## Abstract

**Introduction:**

It is well established that migrants have a 2 to 4 times increase in psychosis risk. However, estimates are highly heterogeneous and vary considerably depending on origin and destination country. It also seems that the relationship between migration and psychosis is complex.

**Objectives:**

In this review, we aim to explore the moderators, mediators and mechanisms behind migration psychosis.

**Methods:**

We searched PubMed using the following terms: “psychosis,” “psychotic,” “migra*”, “immigra*”, “schizophreni*.” “pathogene*”. We limited the search to studies published after 2010 and we screened the title, abstract, and full text. We included a total of 47 studies in this narrative review.

**Results:**

Moderators identified in the literature were country of origin, vitamin D deficiency, male sex, and psychosocial adversity (e.g. exposure to war). Mediators were mostly social, namely discrimination, social exclusion and ethnic minority status, low ethnic density, as well as language distance, unstable housing, and unemployment. Most of the studies we retrieved found that substance use did not fully explain the increased risk for psychosis among migrants. We found that potential pathophysiological mechanisms include stress-induced alterations in dopaminergic neurotransmission, functional and structural alterations in ventral anterior cingulate cortex, as well as possible stress-resultant neuroinflammation.

**Conclusions:**

This review highlights the pathway from psychosocial hardships to neurobiological alterations leading to migration psychosis. Further research is needed to translate these findings into developing preventive measures and tailoring treatment modalities to the migrant population.

**Disclosure of Interest:**

None Declared

